# Community-based interventions for childhood asthma using comprehensive approaches: a systematic review and meta-analysis

**DOI:** 10.1186/s13223-021-00522-9

**Published:** 2021-02-15

**Authors:** Mei Chan, Melinda Gray, Christine Burns, Louisa Owens, Susan Woolfenden, Raghu Lingam, Adam Jaffe, Nusrat Homaira

**Affiliations:** 1 Discipline of Paediatrics, School of Women’s and Children’s Health, Faculty of Medicine, University of New South Wales, Sydney, NSW 2031 Australia; 2 Respiratory Department, Sydney Children’s Hospital, Randwick, NSW 2031 Australia

**Keywords:** Community-based, Meta-analysis, Asthma, Childhood, Comprehensive intervention

## Abstract

**Objective:**

We conducted a systematic review and meta-analysis to determine the effectiveness of comprehensive community-based interventions with ≥ 2 components in improving asthma outcomes in children.

**Methods:**

A systematic search of Medline, Cumulative Index to Nursing and Allied Health Literature (CINAHL), Excerpta Medica Database (EMBASE), Cochrane Library and hand search of reference collections were conducted to identify any research articles published in English between 2000 and 2019. All studies reporting community-based asthma interventions with ≥ 2 components (e.g., asthma self-management education, home environmental assessment or care coordination etc.) for children aged ≤ 18 years were included. Meta-analyses were performed using random-effects model to estimate pooled odds ratio (OR) with 95% confidence intervals (CIs).

**Results:**

Of the 2352 studies identified, 21 studies were included in the final analysis: 19 pre-post interventions, one randomised controlled trial (RCT) and one retrospective study. Comprehensive asthma programs with multicomponent interventions were associated with significant reduction in asthma-related Emergency Department (ED) visits (OR = 0.26; 95% CI 0.20–0.35), hospitalizations (OR = 0.24; 95% CI 0.15–0.38), number of days (mean difference = − 2.58; 95% CI − 3.00 to − 2.17) and nights with asthma symptoms (mean difference = − 2.14; 95% CI − 2.94 to − 1.34), use of short-acting asthma medications/bronchodilators (BD) (OR = 0.28; 95% CI 0.16–0.51), and increase use of asthma action plan (AAP) (OR = 8.87; 95% CI 3.85–20.45).

**Conclusion:**

Community-based asthma care using more comprehensive approaches may improve childhood asthma management and reduce asthma related health care utilization.

## Background

Asthma is a significant public health problem affecting 339 million people worldwide [1]. According to the Global Asthma Report [1], it is the 16th leading cause of disability adjusted life years lost (DALYs) and 28th leading cause of burden of disease.

Despite advancement in understanding of the disease and availability of effective treatments, asthma remains one of the most common causes of preventable visits to emergency department (ED) and admissions to hospital in children [2]. The reasons for poor progress in pediatric asthma control are multifaceted, including difficulties with medication adherence, inadequate asthma education, failure to mitigate environmental triggers, lack of coordination within and between healthcare services etc. [3]. As the roots of these problems often lie outside the acute care system, there is growing interest in developing and implementing an effective community-based approach to improve asthma-related health outcomes in children.

The majority of community-based programs developed in the last decades to improve childhood asthma strategies have mainly focused on single intervention comprising asthma self-management education for the child/caregivers involving only one community settings e.g., school or home. However, reports on the effectiveness of such interventions have not been consistent [4]. In a meta-analysis of 37 studies, it was found that although asthma education alone had significantly reduced the odds of ED visits for asthma, the impact on hospital admissions and urgent physician visits were not significant [4]. This is most likely because asthma is a complex disease with a broad range of contributing factors which are not limited to the physiological, but also include environmental, social and behavioural determinants of health. Therefore, a combination of interventions that address the social behavioural and physiological aspect of asthma through bridging the gap between hospital- and community-based services are necessary to achieve better health outcomes for children with asthma. Comprehensive asthma programs with multicomponent interventions that include self-management skills, environmental triggers avoidance, care coordination, advocacy for community or governmental support etc., and various members of the community e.g., schools, neighbourhood, government/policy makers etc., have been developed and implemented. However, a critical review of recent evidence and effort to quantify the effectiveness of these more comprehensive community-based approaches which interconnect different stakeholders is lacking.

## Objectives

The objectives of this systematic review were to systematically evaluate the existing body of evidence to identify the key components of multicomponent interventions and determine their effectiveness in improving health outcomes in children with asthma.

## Methods

This systematic review adopted the Preferred Reporting Items for Systematic Reviews and Meta-analyses (PRISMA) standards (Prospero registration number: CRD 42019133776).

### Types of studies

We included randomized clinical trials (RCTs) and quasi-experimental studies (i.e., pre- and post- intervention studies, retrospective cohort studies) in this review.

### Types of participants

Only studies including children aged ≤ 18 years, who have been diagnosed with asthma or shown symptoms of asthma, and participated in a community-based asthma program with multiple intervention components were considered in this review.

### Types of interventions

Studies selected involved comparison of community-based interventions with ≥ 2 components that aimed to improve health outcomes or management pathways versus no intervention for children with asthma. The interventions could have taken place in a variety of community settings e.g., homes, community health clinics, schools etc., and carried out by any health care professionals (e.g., nurses, asthma educators, social workers or community health workers etc.). Interventions included (A) asthma self-management education, (B) home environmental assessment (i.e. home visits for trigger assessments with or without remediation supplies), (C) care coordination (i.e., connecting patients/families with relevant health care or social services), (D) school involvement (e.g. asthma education for patients or school personnel, behavioural counselling at school etc.), (E) involvement of primary healthcare providers for ongoing asthma assessment, provision of AAP etc., (F) community involvement (e.g. awareness campaign, neighbourhood support etc.) or (G) advocacy for government/local organization involvement in policy changes.

### Types of outcome measures

Outcomes of interest included differences between those with and without intervention in (1) acute care utilization e.g., ED visits and hospitalization, (2) asthma control e.g., asthma symptom day/night, use of asthma action plan (AAP), asthma medication uses or Asthma Control Test (ACT) score, (3) pulmonary function e.g., forced expiratory volume in one second (FEV_1_), and (4) productivity e.g., school or work absenteeism. Qualitative/narrative data collected from interviews or open questions were not analyzed.

### Search methods for identification of studies

Eligible articles were identified from four electronic databases: Medline, Cumulative Index to Nursing and Allied Health Literature (CINAHL), Excerpta Medica Database (EMBASE) and Cochrane Library. Additional studies were retrieved from the reference lists of identified articles. Original articles published between 2000 and 2019 were searched using a combination of MeSH keywords: (1) “asthma”; (2) “child” or “pediatrics” or “childhood”, “child health”; (3) “community” or “community care” or “community health service”, “public health”, “multicomponent”, “integrated care”, “comprehensive”, “collaborative”, “care coordination”. Studies were included if they were: (1) published in English language, (2) available in full-text, (3) conducted in humans, (4) conducted in children with asthma, (5) involving community-based interventions. Studies were excluded if they were: (1) review articles, (2) unpublished data, or (3) qualitative/narrative research studies.

### Study selection process

**Fig. 1:**
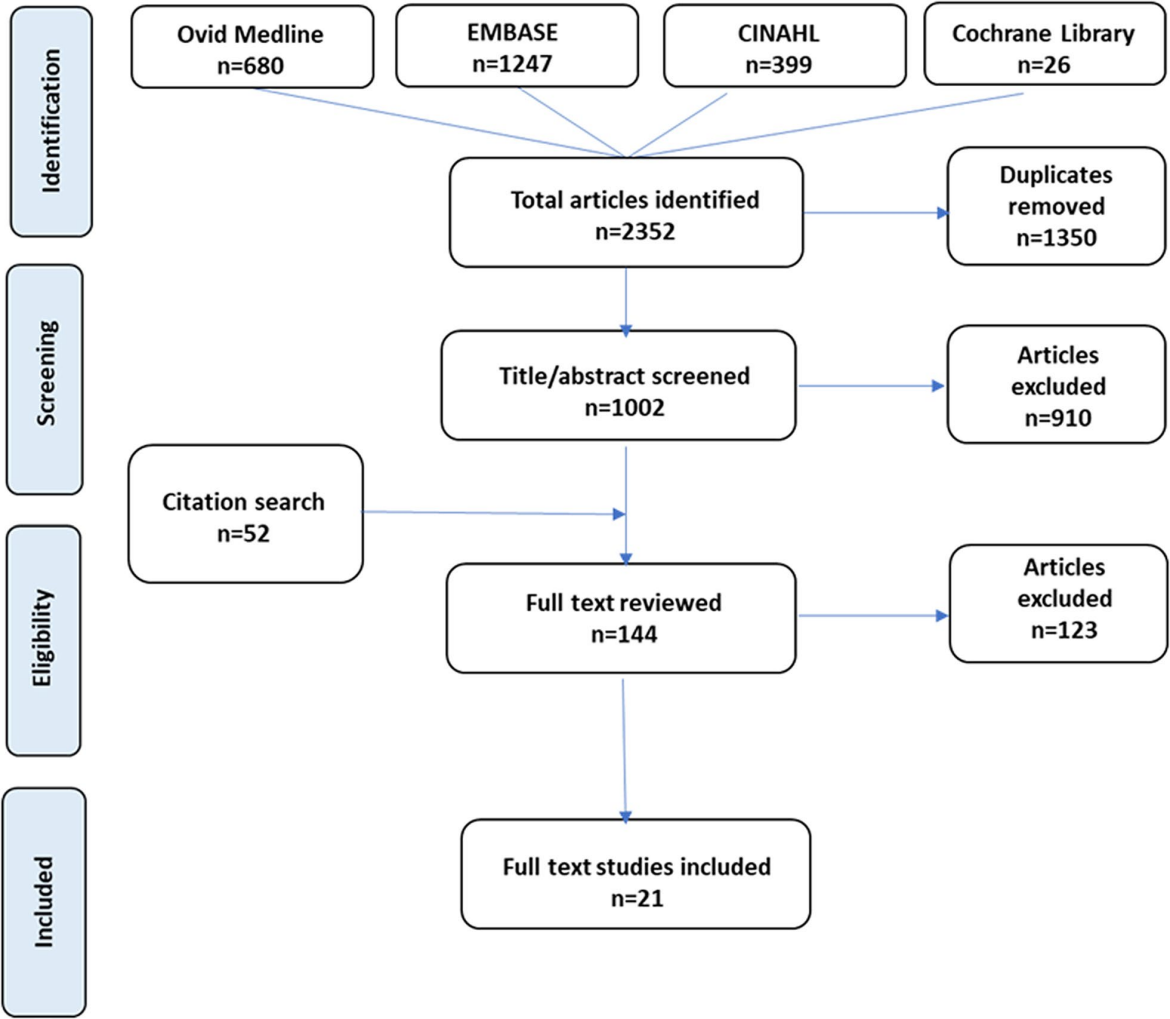
Preferred Reporting Items for Systematic Reviews and Meta-Analyses (PRISMA) flow diagram. Footnote: *CINAH*L Cumulative index to nursing and allied health literature; *EMBASE* Excerpta Medica Databases

Figure 1 summarizes details of the study selection process. All articles identified through database searches were screened for duplication, full text availability and language used. Titles and abstracts of the remaining articles were reviewed for eligibility by one reviewer. Studies with titles or abstracts that met the inclusion criteria had full-text review. A research team member assessed the contents of the articles to determine whether they were to be included in this review and final decision was reached after agreement was obtained from the principal investigator.

### Data extraction and quality assessment

The following data were extracted from the selected studies and entered into a table developed by the research team: authors, year of study, study duration, study design, sample size, age, care provider, ethnicity, asthma severity, intervention components and key findings. Accuracy and completeness of the data were counter checked by the second reviewer.

The strength and quality of the studies was assessed by using the Risk of Bias In Non-randomized Studies (ROBINS-I) tool developed by the Cochrane Bias Method Group [5]. The strength of the evidence was assessed based on seven domains: (1) bias due to confounding, (2) bias in selection of participants into the study, (3) bias in classification of interventions, (4) bias due to deviations from intended interventions, (5) bias due to missing data, (6) bias in measurement of outcomes, (7) bias in selection of the reported result [5].

### Data analysis

Meta-analyses were performed to pool data from studies with sufficient information of the same outcome measures. Dichotomous data were analyzed using odds ratio (OR) and continuous outcomes were analyzed using weighted mean difference (MD). DerSimonian and Laird’s random effects model was used to estimate the overall effect size with 95% confidence interval (CI). For data that were unavailable or could not be extracted from information provided, the authors of the studies were contacted to obtain the relevant information. For continuous data, missing standard deviations were estimated from other summary statistics (e.g., confidence intervals, standard errors, *t* values or *p* values) using the methods described by Higgins and Green [6].

Standard chi-squared (*X*^*2*^) test and *I*^*2*^ statistics was calculated to evaluate the level of heterogeneity of the pooled results (i.e., whether the variance was genuine or due to sampling error), and considerable heterogeneity was present if *I*^*2*^ ≥ 75% and *X*^*2*^ ≤ 0.1 [7]. Sensitivity analysis was conducted using stepwise single-study elimination method to determine if our decision had any impact on the pooled results by omitting each study. All statistical analyses were carried out using Review Manager (RevMan) software version 5.3 [8].

## Results

### Results of the search

A total of 2352 potential articles were identified from the initial literature search. After duplicates were removed, titles/abstracts of 1,002 articles were screened for eligibility and full text availability. Of these, 92 articles, together with the 52 articles retrieved from the reference lists, underwent full text review. As a result, a total of 21 studies met the selection criteria and were included in this systematic review. Details of the search strategy is depicted by a PRISMA flow chart in Fig. 1.

### Study characteristics

**Fig. 2:**
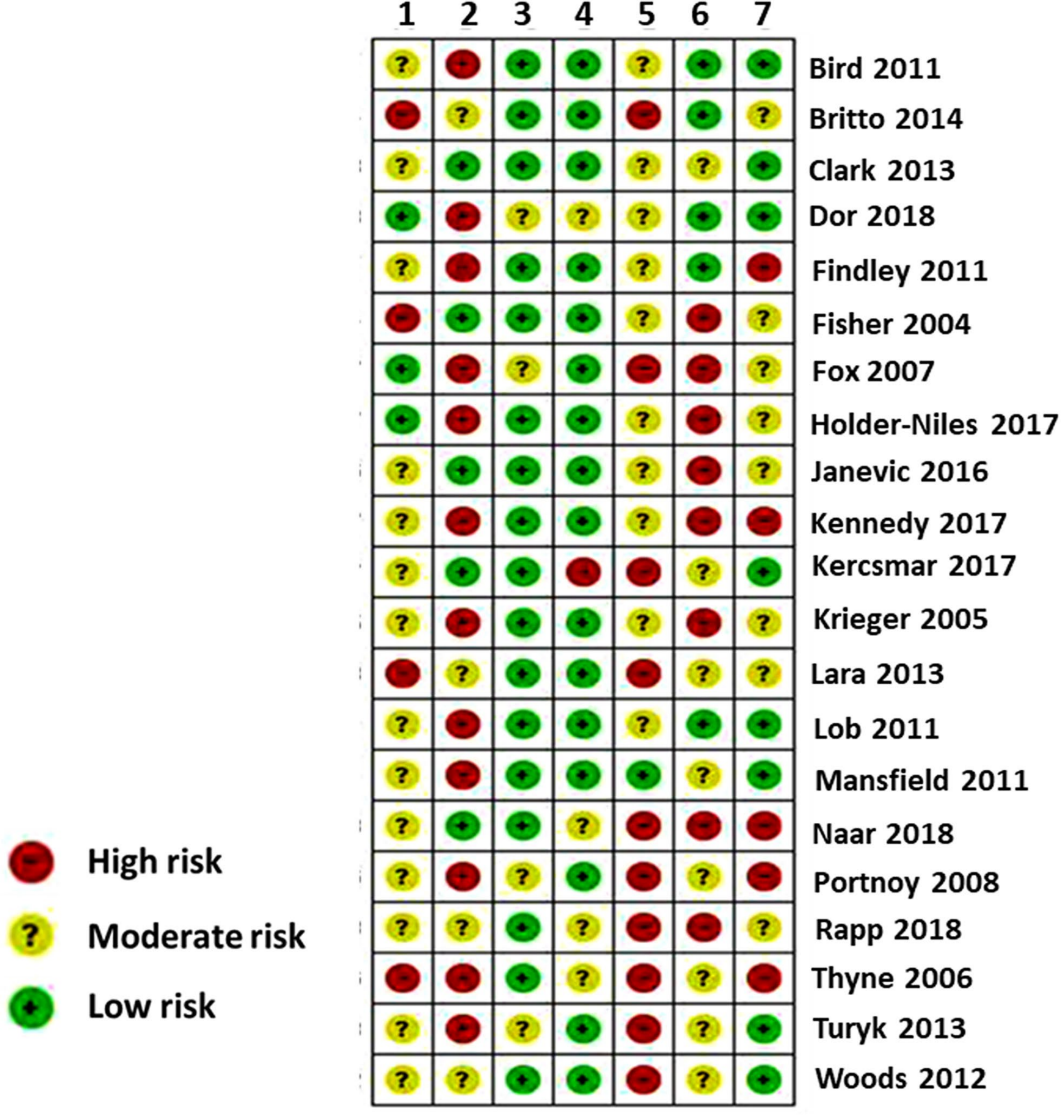
Summary of risk of bias of included studies. Legends: 1 = Bias due to confounding. 2 = Bias in selection of participants into the study. 3 = Bias in classification of interventions. 4 = Bias due to deviations from intended interventions. 5 = Bias due to missing data. 6 = Bias in measurement of outcomes. 7 = Bias in selection of the reported result

Among the 21 studies, nineteen were pre-post intervention studies [9–27], one was RCT [28] and one was retrospective cohort [29] study. Overall, risk of bias was moderate as illustrated in Fig. 2. All of the studies were carried out in the United States (US), except one in Australia [10]. Ethnic minorities (i.e. African American and Hispanic American) were the focus in 18 of the 20 studies [9, 11, 12, 14–18, 20–29]. Sample sizes varied widely ranging from 65 to 134,480 children (median = 295) aged from 0–18 years (Table 1). Interventions were delivered mainly by nurses including asthma educators, nurse practitioners etc. (13/21), [9–14, 16, 18–20, 22, 23, 25] followed by community health workers (11/21), [12, 14–17, 20–22, 25, 27, 29] social workers (4/21), [11, 13, 24, 28] physicians (3/21), [11, 22, 25] respiratory therapists (2/21), [13, 15] psychologists (1/21), [28] pharmacist (1/21) [13].

**Table 1:** Characteristics and key findings of studies included

Author (year)	Study design	Study duration	Sample size	Age	Care provider	Ethnicity of study population	Asthma severity	Interventions components	Key findings
Naar et al. [28]	RCT	1 year	167	12–16 years old; mean = 13 years	Psychologists, social workers	African American	Moderate Severe	A, B, C, D	ED visits: NS; Hospitalizations: decreased by 72% in intervention group vs 48% in control; FEV_1_: improved by 9.8% in intervention group vs 4.5% in control; Frequency of asthma symptoms: decreased by 48% in intervention group vs 3.4% in control
Kennedy et al. [9]	Pre-post intervention	1 year	590	5–12 years old; mean = 7.8 years	Nurses	African American = 9%; Hispanic = 83%; Others = 8%	Moderate Severe	A, B, C	Urgent visits: intervention group = 53% vs control = 74%; Hospitalizations: intervention group = 5%, control = 14%; Asthma symptom days: mean change in intervention group = -3.3 days, control = -2.28 days (p < 0.001); Short-acting medication uses: intervention group = 32%, control = 62% (p < 0.01)
Bird et al. [10]	Pre-post intervention	3 years	295	1–18 years old; mean = 5.3 years	Nurses, asthma educators, asthma care facilitators	Unspecified	Not specified	A, B, C, E	ED visits: decreased by 57% in intervention group vs NS change in control; Hospitalizations decreased by 74% in intervention group vs NS change in control; PAQLQ significantly improved in intervention group: activity limitation scores increased by 5.6 units, emotional function scores increased by 9.1 units
Clark et al. [29]	Retrospective cohort	5 years	134,480	2–18 years old	CHWs	African American = 62–64%; Caucasian = 7–15%	Not specified	A, B, C, D, E, F, G	Hazard of having a ED visit, hospitalization and urgent care visit during the study period was 6%-7% higher (p < 0.01–0.02) for children in comparison group than those with intervention
Holder-Niles et al. [11]	Pre-post intervention	2 years	141	4–15 years old; mean = 13.4 years	Physicians, nurse practitioners, asthma educators, social workers, community resource specialists, research assistants	African American = 62%; Hispanic = 14%; Caucasian = 3%; Others = 22%	Not specified	A, B, C, D, E	ED visits: decreased by 63%; Urgent care visits: decreased by 40%; Hospitalizations: decreased by 69%
Janevic et al. [12]	Pre-post intervention	1 year	1223	0–18 years old	Nurses, health educators, CHWs	African American = 50.4%; Hispanic = 42.1%; Caucasian = 1.7%; Others = 5.3%	Severe	A, B, C, D, E, F, G	ED visits: decreased by 63%; Hospitalizations: decreased by 69%; Symptom days: decreased by 2.2 days/month; Symptom nights decreased by 1.9 nights/month
Kercsmar et al. [13]	Pre-post intervention	6 years	36,000	2–17 years old	Physicians, social workers, respiratory therapists, nurses, care coordinators, pharmacists	Unspecified	Not specified	A, B, C, D, E, G	ED visits: decreased by 42.4%; Hospitalizations: decreased by 41.8%
Lara et al. [14]	Pre-post intervention	1 year	117	Mean age = 5 years	CHWs, community nurses, physicians, environmental counsellors	Hispanic	Moderate Severe	A, B, C	ED visits: decreased by 45%; Hospitalizations: decreased by 62%; Symptom days: decreased by 72%; Symptom nights: decreased by 68%; Asthma symptom scores: decreased by 38%; Short-acting medication uses: decreased by 76%
Lob et al. [15]	Pre-post intervention	2 years	4049	0–18 years old; mean = 7.4 years	Health educators, CHWs, medical assistants, respiratory therapists	African American = 11%; Hispanic = 77%; Caucasian = 6%; Others = 6%	Severe	A, B, C, D, E, F, G	ED visits: decreased by 68%; Hospitalizations: decreased by 69%; Symptom days: decreased by 73%; Symptom nights: decreased by73%; Night-time symptoms decreased by 67%; Short-acting medications uses decreased by 67%; School absenteeism: decreased by 53%; work absenteeism decreased by 55%
Mansfield et al. [16]	Pre-post intervention	1 year	724	0.6–19 years old; mean = 8 years	Nurses, health educators, CHWs	African American = 50%; Hispanic/others = 37%; Caucasian = 13%	Moderate Severe	A, B, C, D, E, F, G	ED visits: decreased by 51%; Hospitalizations decreased by 55%; Symptom days: decreased by 56%; Symptom nights: decreased by 55%; Days with rescue med use decreased by 1.91 days; School absenteeism: decreased by 57%
Rapp et al. [17]	Pre-post intervention	1 year	187	2–18 years old; mean = 5 years	Asthma educators, CHWs	African American = 88%; Caucasian = 7%	Not specified	A, B, C, D, E, F, G	ED visits: decreased by 46%; Hospitalizations: NS; Short-acting medication uses: NS; Asthma symptoms: improved significantly
Woods et al. [18]	Pre-post intervention	1 year	283	2–18 years old; mean = 7.9 years	Nurses	African American = 40%; Hispanic = 52%	Not specified	A, B, C, E, F, G	ED visits: decreased by 68%; Hospitalizations: decreased by 84.8%; Activity limitation days: decreased by 56%; School absenteeism: decreased by 41%; Work absenteeism: decreased by 49.7%
Dor et al. [19]	Pre-post intervention	1 year	570	5–12 years old	Nurses	Unspecified	Not specified	A, B, C	Urgent care visits: mean change = -0.405 visits (p < 0.001); Hospitalizations: mean change = -0.305 (p < 0.001); Symptom-free days: mean change = 6.319(p < 0.001); Probability of being short-acting asthma medication user decreased by 19.1%; Probability of being long acting asthma medication user increased by 30%; School absenteeism: mean changes = -0.973 days (p < 0.001)
Findley et al. [20]	Pre-post intervention	1 year	724	0–18 years old	Nurses, health educators, CHWs	African American & Hispanic	Not specified	A, B, C, D, E, F, G	ED visits: decreased by 49%; Hospitalizations: decreased by 45%; School absenteeism: decreased by 83%; 81% obtained AAP from health professional post-intervention; 73% reporting daily controller use post-intervention
Fisher et al. [21]	Pre-post intervention	1 year	249	5–14 years old; mean = 9.5 years	CHWs	African American	Not specified	A, B, C, D, E, F, G	Acute care visits: NS; Index of Asthma Attitudes: NS; Asthma management index: NS
Fox et al. [22]	Pre-post intervention	2 years	560	5–18 years old; mean = 10.1 years	CHWs, physicians, nurse practitioners, nurses	African American = 14.3%; Caucasian = 2.2%; Hispanic = 81.7%; others = 1.7%	Moderate Severe	A, B, C, D, E, F, G	ED visits: decreased by 68%; Acute care visits: decreased by 64%; Hospitalizations: decreased by 80%; Symptom days: decreased by 69%; Symptom nights: decreased by 83%; Short-acting medication uses: decreased by 72%
Britto et al. [23]	Pre-post intervention	4 years	322	Teenagers (age range unspecified)	Nurses, nurse practitioners	African American	Moderate Severe	A, C, F, G	ED visits: NS; Hospitalizations: NS; ACT score: proportion of patients with ACT score 25 increased from 10 to 30%
Portnoy et al. [24]	Pre-post intervention	1 year	170	5–12 years old	Social workers	African American = 70%; Caucasian = 12%; Hispanic = 12%; Others = 4%	Moderate Severe	A, B, C, D, F	ED visits: NS; Hospitalizations: NS; AAP uses: increased by 34%; Controller medication uses: increased by 12%
Thyne et al. [25]	Pre-post intervention	2 years	65	0–18 years old; mean = 7.3 years	CHWs, physicians, nurse practitioners	African American = 43%; Hispanic = 43%; Asian = 11%; Others = 3%	Not specified	A, B, C, E, F, G	Symptom days: decreased by 45%; Symptom nights: decreased by 46%; Activity limitation days: decreased by 39%; FEV1: NS; Controller medication uses: increased by 127%; AAP uses: increased from < 1% to 100% (p < 0.001)
Turky et al. [26]	Pre-post intervention	1 year	218	0–17 years old	Community health educators	African American	Not specified	A, B, C, D, E, F, G	ED visits: decreased by 49%; Urgent care visits decreased by 58%; Hospitalizations: decreased by 71%; School absenteeism: decreased by 70%; Work absenteeism: decreased by 64%
Krieger et al. [27]	Randomized controlled trial	1 year	274	4–12 years old; mean = 7.4 years	CHWs	African American = 31.9%; Hispanic = 17.4%; Caucasian = 12.3%; Vietnamese = 25.4%; Asian = 9.4%; Others = 3.6%	Not specified	A, B, C, F, G	Urgent visits: decreased by 15%; Symptom days: decreased by 4.7 days; School absenteeism: NS; Work absenteeism: NS; Short-acting medication uses: NS

A = Asthma & self-management education; B = Home environmental assessment; C = Care coordination; D = School involvement; E = Primary provider involvement; F = community awareness; G = Government/organization involvement

*AAP* Asthma action plan, *FEV*_*1*_ Forced expiratory volume in one second, *ACT* Asthma Control Test score, *PAQLQ* Pediatric Asthma Quality of Life Questionnaire, *ED* Emergency Department, *NS* Not significant

As shown in Table 1, intervention components of each programs were different. However, education on basic asthma knowledge and self-management skills, care coordination to connect patients/families with relevant primary care or social services, and home visit for environmental trigger assessment were common intervention elements (i.e., core interventions) across 20 of 21 reviewed studies. Other interventions such as provision of environmental remediation products e.g. allergen-proof beddings and pillow encasings, cleaning supplies, cockroach abatement etc. were employed in 13 studies [9, 11–14, 16–21, 26, 27]. Engagement with schools to increase asthma awareness, provide asthma education to staff members, promote asthma-friendly school environment or improve communication between care providers and schools, were parts of the intervention programs in 13 of the 21 studies [11–13, 15–17, 20–22, 24, 26, 28, 29]. Fourteen studies [10–13, 15–18, 20–22, 25, 26, 29] also worked with primary care providers to address issues of healthcare accessibility, inform of patient’s progress, perform medical assessment, medication adjustment, review of AAPs, or update with practice change and latest asthma guidelines. Whereas, in 14 studies, [12, 15–18, 20–27, 29] effort was made through community fairs or education sessions to increase community awareness and social support for children with asthma. Additionally, public or community organizations/agencies were involved in 14 studies [12, 13, 15–18, 20–23, 25–27, 29] to bring about changes in clinical or community systems to ensure more effective disease management.

### Effect of interventions on health care utilizations

**Fig. 3:**
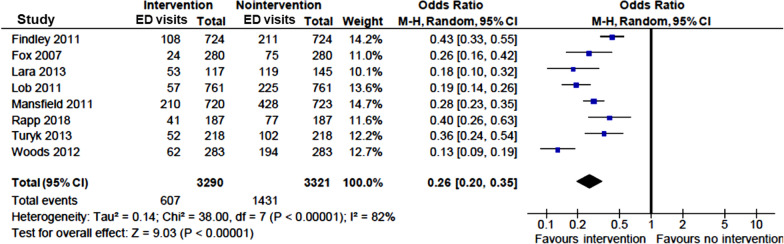
Pooled odds ratio (OR) of asthma-related ED visits in children with vs without community-based intervention

Overall, ED visits were evaluated in 16 studies, [10–18, 20, 22–24, 26, 28, 29] of which, 13 reported a significant post-intervention reduction in asthma-related ED visits [10–18, 20, 22, 26, 29] (Table 1). Among these 13 studies, eight presented complete dichotomous data on ED visits (yes/no). Meta-analysis of these studies revealed that ED visits were significantly reduced after multicomponent community-based interventions (OR = 0.26; 95% CI 0.20–0.35) (Fig. 3).

**Fig. 4:**
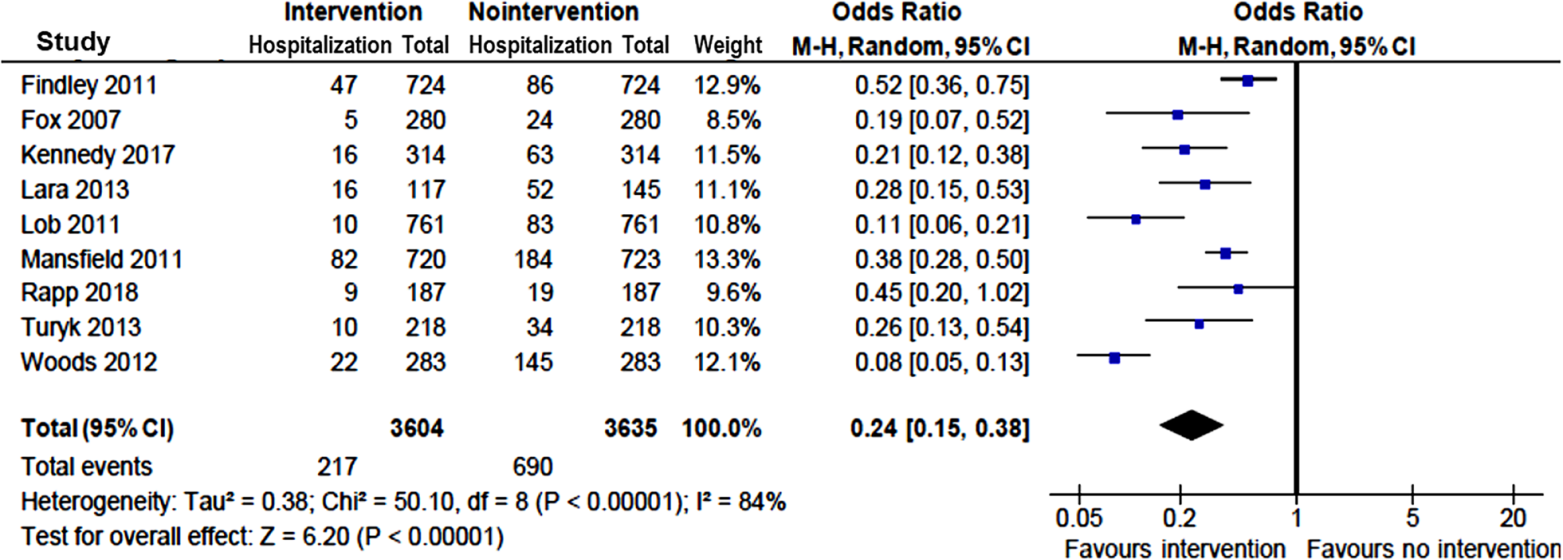
Pooled odds ratio of hospitalizations in children with vs without community-based intervention

Multicomponent interventions were also associated with reduction in asthma-related hospitalizations in 15 [9–16, 18–20, 22, 26, 28, 29] of 18 studies [9–20, 22–24, 26, 28, 29] that reported the outcome measure (Table 1). The odds of asthma-related hospitalization was reduced by 76% in children with vs without interventions (OR = 0.24; 95% CI 0.15–0.38) (Fig. 4)**.**

### Effects on asthma control

**Fig. 5:**
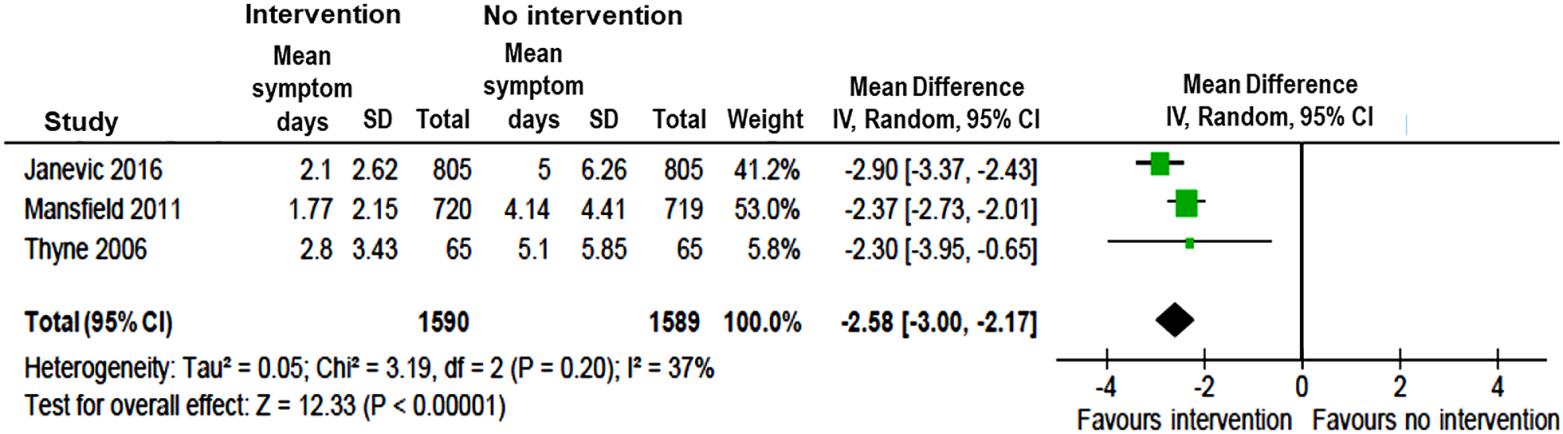
Pooled estimates of mean difference for days with asthma symptoms in children with vs without community-based intervention

**Fig. 6:**
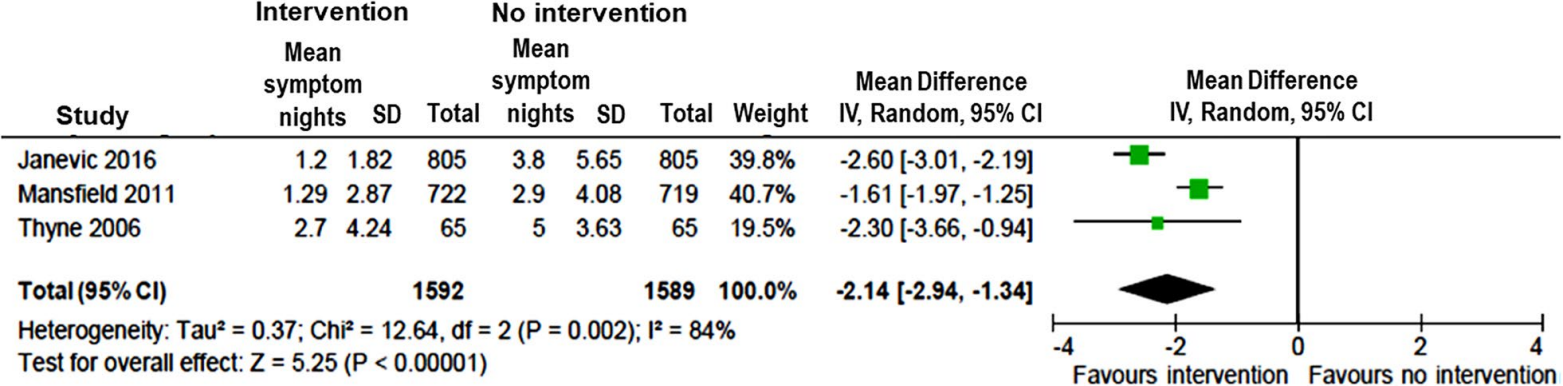
Pooled estimates of mean difference for nights with asthma symptoms in children with vs without community-based intervention

Ten studies measured changes in asthma symptoms from pre- to post-implementation of intervention programs [9, 12, 14–17, 19, 22, 25, 28]. All 10 studies reported a beneficial effect of intervention with significant decrease in days with asthma symptoms, while six of the studies [12, 14–16, 22, 25] also reported decrease in nights with asthma symptoms (Table 1). The pooled intervention effects, in terms of mean differences (MDs), were − 2.58 days per fortnight (95% CI − 3.00, − 2.17) (Fig. 5) and − 2.14 nights per fortnight (95% CI − 2.94, − 1.34) (Fig. 6) with asthma symptoms based on data available in three studies. [12, 16, 25]

Two studies [25, 28] measured lung function of the study population, in terms of FEV_1_, however, only one of them [28] reported significant improvement_,_ of 15.6% (i.e., from 2.05 at baseline to 2.31 at 12 months; *p* < 0.001). One study assessed ACT score and reported an increase in the proportion of patients with optimally well-controlled asthma (i.e., ACT score of 25) from 10 to 20% 2 years after interventions [23] (Table 1).

**Fig. 7:**
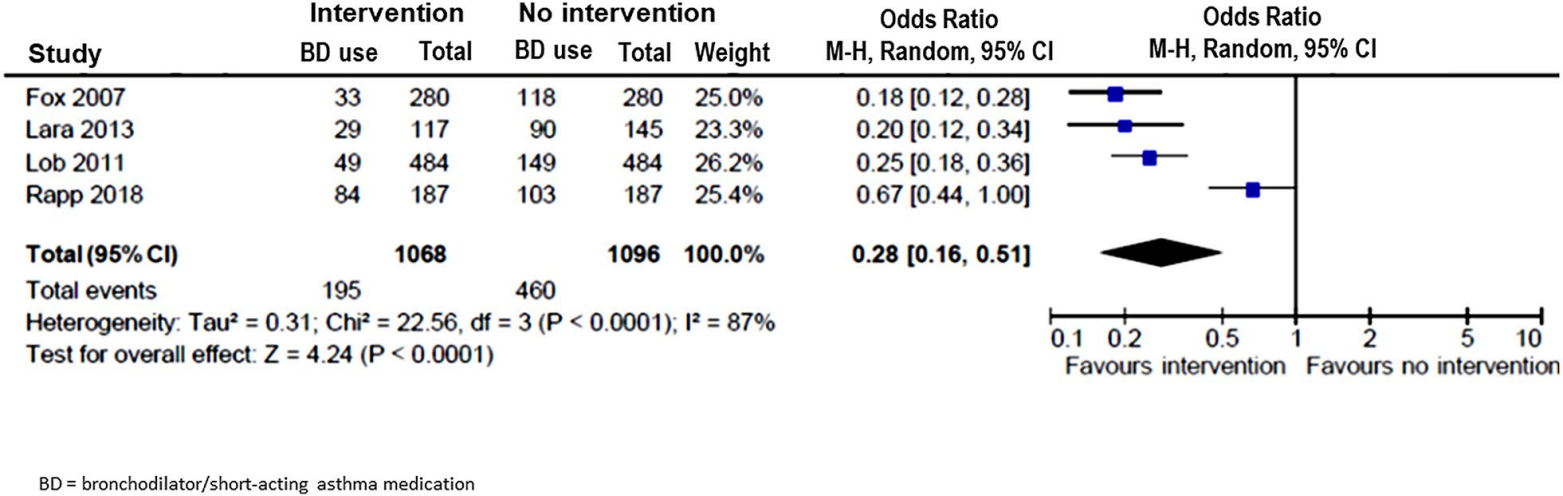
Pooled odds ratio (OR) of short-acting asthma medication/bronchodilator (BD) uses in children with vs without community-based intervention

**Fig. 8:**
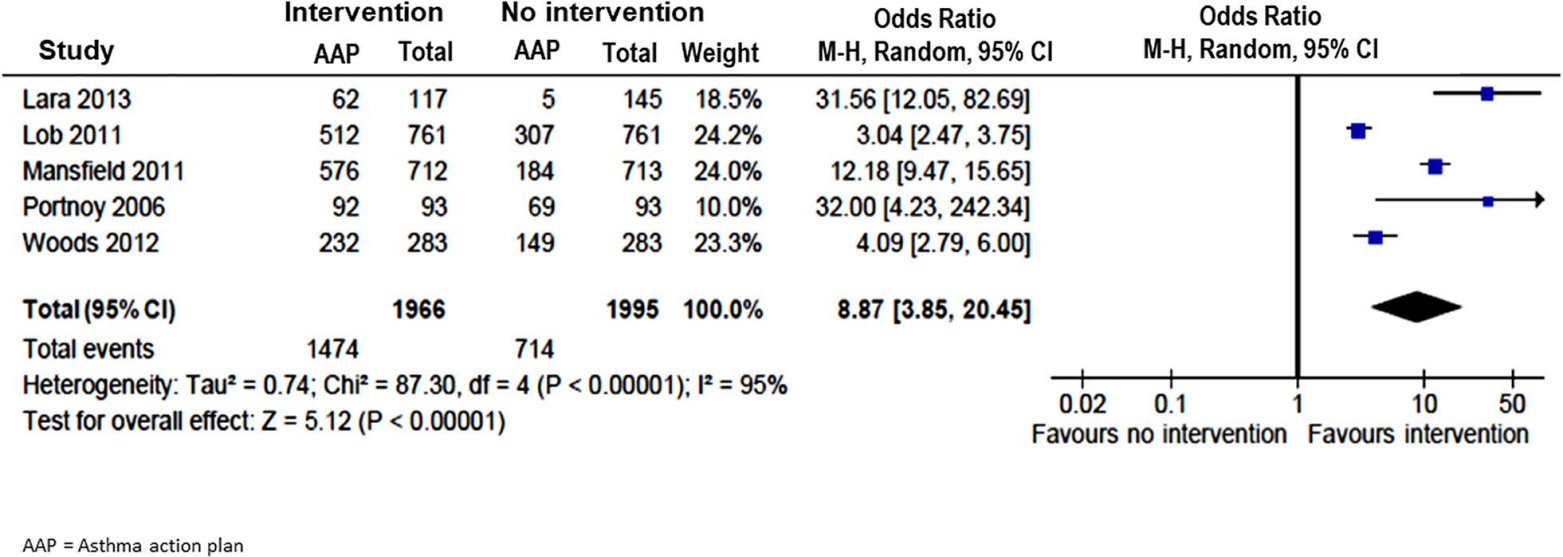
Pooled odds ratio (OR) of asthma action plan (AAP) uses in children with vs without community-based intervention

Seven studies [9, 14–17, 19, 22] measured the use of short-acting asthma medications or bronchodilators (BD) e.g., β-2 agonists. Six [9, 14–16, 19, 22] reported a significant decrease in the number of patients needing these medications (Table 1). Meta-analysis on the four studies with complete data [14, 15, 17, 22] showed a significant reduction in need for short-acting asthma medications/BD with interventions (OR = 0.28; 95% CI 0.16–0.51) (Fig. 7). Furthermore, use of AAP was also found to be significantly increased in five studies [14–16, 18, 24] after interventions (OR = 8.87; 95% CI 3.85—20.45) (Fig. 8).

### Effect on other outcomes

Use of long-acting/controller asthma medication was evaluated in four studies and was found to have increased by 12% to 127% (19, 20, 24, 25) (Table 1). Data on days missed from school were insufficient for meta-analysis. However, all the six studies [15, 16, 18–20, 26] that reported asthma-related school absenteeism found a significant reduction, ranging from 41 to 83%. Three of these six studies [15, 18, 26] also reported reduction (50–64%) in days missed from work by parents/carers due to their child’s asthma (Table 1).

## Discussion

Our comprehensive systematic review and meta-analysis involving a total of 181,408 children with asthma has demonstrated that community-based asthma interventions with multiple components provided at various community settings can reduce health service use, improve asthma-related symptoms and potentially reduce school absence. We have identified that almost all (95%) community-based multicomponent interventions included at least three main components i.e., self-management asthma education, home environmental risk assessment and care coordination.

As asthma cannot be cured, focus of asthma management need to be placed in measures to achieve optimal asthma control and minimizing risk of severe complications. Like many other chronic illnesses, effective asthma management requires involvement of multiple stakeholders i.e. family, school, community and policy organizations or agencies, to support the patient’s role as manager of his/her own health [30, 31]. In the Chronic Care Model (CCM), Wagner and colleagues [30] identified six elements (i.e. self-management support, community resource, decision support, delivery system design, organizational support, clinical information system) to overcome the deficiencies in chronic disease management such as poor adherence to practice guidelines, lack of care coordination and planned care, lack of active follow up and inadequate training for patients to manage their illness. Of the six elements of CCM, self-management is the key component which involves recognizing the patient’s central role in managing their illness, and provide patients and families with support to acquire skills and confidence in managing their own health care [31]. This is of particular importance in asthma care as evident from our systematic review where education on basic asthma knowledge and self-management skills was one of the main core interventions. However, intervention program with only asthma education may not be adequate for achieving optimal asthma control. In a recent study of a community outreach program which focused on asthma education for Aboriginal children with asthma, the investigators had failed to find significant improvement in unscheduled medical visits and quality of life despite some improvement in carer’s knowledge and skills about asthma [32]. A review conducted by Wu & Takaro [33] demonstrated that a combination of interventions, involving both environmental measures for trigger reduction and education of self-management skills, was effective in improving clinical symptoms and acute service utilizations for asthmatic children, but such improvements were not observed in studies with asthma education as the only intervention. Several systematic reviews have also been conducted previously on educational programs for asthma self-management but the results were inconsistent [4, 34–36], suggesting that asthma education alone may not be sufficient for effective asthma management, especially for children.

Home visit and environmental trigger assessment is another commonly used interventions among studies included in this review. It is well-documented that asthma exacerbations are linked to indoor housing conditions and exposure to allergens such as inadequate ventilation, environmental tobacco smoke, pests etc. [37]. Home visitations provide an opportunity to assess and address potential environmental triggers that caused asthma exacerbation. Many studies have evaluated the impact of interventions targeting asthma triggers in the home environment. Although improvement in asthma symptoms and urgent care visits were consistently reported across the studies, level of asthma control and hospital admissions due to asthma were uncertain [38–40]. Furthermore, families receiving home-based interventions were often supplied with environmental remediation products (e.g. vacuum cleaners, bed coverings, pest abatements) to eliminate potential triggers in the home environment. It has been suggested that the provision of environmental remediation products may not provide additive benefit to asthma outcomes [41]. This is somewhat consistent with our findings. When comparing between studies with and without environmental remediation supplies, there was no additional improvement observed in both ED visits and hospitalizations associated with asthma [17, 21].

Care coordination is a crucial component in the effort to assist patients or families in navigating through health care or social systems. It facilitates interaction between patients/families and the health care providers, as well as linkage to social services to address family needs or problems. In all the asthma programs reviewed here, care coordination is one of the core interventions together with self-management education and home environmental assessment. The three reviewed studies [12, 16, 20] that placed specific emphasis on care coordination consistently reported significant reduction in ED visits and hospital admissions, which is indicative of the importance of care coordination in improving health outcomes for children with asthma. However, whether care coordination alone is adequate to achieve effective asthma control has yet to be determined and should be the subject for future research investigations.

Apart from the core interventions (i.e., self-management education, home environmental assessment and care coordination), most of the reviewed studies had their interventions extended to schools, primary practices, public/organizational agencies and the wider community to promote asthma awareness and support, as well as to advocate for more asthma-friendly environment or policy changes. Although nine studies had similar program designs that included all seven intervention elements (i.e. all three core components plus the involvement of schools, primary care providers, community campaigns, policy makers/organizations) [12, 15–17, 20, 21, 29], there were great variations between the remaining 12 studies in regarding the components of their interventions. Therefore, it is not possible to clearly identify the optimal combination of intervention elements that had led to the improvement observed in the summary effects.

It was also worth noting that none of the studies included the use of telehealth care, which has drawn considerable attention in the recent pandemic outbreak. In general, telehealth refers to health services delivered by electronic communication media such as telephone, video or internet. It is effective in overcoming geographical barriers and has been shown to be cost-effective in providing health advice and education for patients with chronic illnesses [42]. However, a recent systematic review of 21 RCTs using telehealth care (including text messaging, telephone, video conferencing etc.) did not show any significant improvement in quality of life or ED visits for patients with asthma [43]. More researches are needed to confirm the impact of telehealth on asthma management for children.

Several limitations of this review should be noted. There are few RCT studies available for community-based interventions with multiple components, in particular, for childhood asthma. We only identified one RCT in this review. Comparisons in our meta-analyses were made primarily between before and after intervention, which may lead to overestimation of the summary effect size. However, the effects of studied interventions were, in general, consistent and followed the same positive trend across the measured outcomes. In addition, in the six studies [9, 10, 19, 21, 28, 29] that included comparator groups, their findings were in agreement with that of the meta-analysis, with five out of the six studies [9, 10, 19, 28, 29] reported greater improvements in asthma outcomes among intervention groups than the controls.

High level of heterogeneity was identified and was likely due to different study designs, settings and intervention components, as well as low number of studies included. In order to incorporate the between-study variations, random effect models were used to estimate the summary effect size. Also, we performed subgroup analyses to explore the heterogeneity amongst studies and our results showed little change to the direction or magnitude of the estimated effects. Meanwhile, with stepwise single-study elimination analysis, we did not observe significant change in the outcomes for which pooled effect sizes were calculated, suggesting that there was minimal publication bias.

Although a considerable number of key words and search terms have been used, it is possible that we did not extract all the relevant articles in this review. Thus, in addition to electronic searches, we conducted hand search of reference lists to extract all relevant articles in the existing literatures. Most of the studies included were conducted in the US, with majority of the participants from ethnic minority background and low-income households. As healthcare system in US is very different from other countries, results of this review may not be generalizable. Finally, we included only articles written in English language and might have missed findings from studies published in other languages.

## Conclusion

Asthma management in children is complex and requires coordinated efforts from a wide range of stakeholders. To enable effective disease management, it needs engagement of community and collaboration between families, schools, primary care providers and government/organizational agencies. As summarized by Clark et al. [44], a successful asthma program should be “community-centred”, “clinically connected” and “continuously collaborative”. Community-based asthma programs with comprehensive approach examined in the present review aligned with these guiding principles and could be an effective model of care in improving health outcomes and reducing acute care need for children with asthma.

## Data Availability

All data generated or analysed during this study are included in this published article.

## Abbreviations

CINAHL: Cumulative index to nursing and allied health literature
EMBASE: Exerpta Medica dataBASE
ED: Emergency department
AAP: Asthma action plan
RCT: Randomised controlled trial
OR: Odds ratio
MD: Mean difference
DALY: Disability adjusted life year
ACT: Asthma control test
FEV^1^: Forced expiratory volume in one second
ROBINS-I: Risk of bias in non-randomised studies tool
CCM: Chronic care model
